# Difference in Tableting of Lubricated Spray-Dried Mannitol and Fluid-Bed Granulated Isomalt

**DOI:** 10.3390/pharmaceutics17121566

**Published:** 2025-12-04

**Authors:** Valentyn Mohylyuk, Kirils Kukuls, Alīna Jaroslava Frolova, Zoltán Márk Horváth, Tetiana Kolisnyk, Elżbieta Maria Buczkowska, Līga Pētersone, Adrien Pelloux

**Affiliations:** 1 Leading Research Group, Faculty of Pharmacy, Riga Stradiņš University, LV-1007 Riga, Latvia; 2 School of Pharmacy, Queen’s University Belfast, Belfast BT7 1NN, UK; 3 Application Laboratory, Medelpharm, 01700 Beynost, France

**Keywords:** tablet diluents, compaction simulation, ejection pressure, mannitol, isomalt, residual die-wall pressure, friction coefficient

## Abstract

**Background**: Polyols are widely used as tablet diluents due to their high solubility, favourable taste, and ability to form robust tablets. Thus, commercially available polyols, such as mannitol and isomalt, can be considered for the preparation of low-drug-dose formulations with a high polyol load. **Methods/Results**: This study investigated spray-dried mannitol (Mannogem^®^ XL Opal SD and Pearlitol^®^ 200 SD) and fluid-bed granulated isomalt (galenIQ™ 720 and galenIQ™ 721) at magnesium stearate levels of 0.5 and 3.0 wt.% and consolidation pressures of 100 and 300 MPa. During the tableting of 100 consecutive tablets, materials displayed different ejection force profiles: galenIQ™ 720 and galenIQ™ 721 demonstrated low and stable ejection pressures; Mannogem^®^ displayed a lubricant- and compaction pressure-dependent profile, whereas Pearlitol^®^ produced the highest ejection forces, particularly at 0.5 wt.% magnesium stearate. To elucidate these differences, the used materials were characterised in terms of SEM imaging, moisture content, surface area and porosity analysis, particle size distribution, pXRD, and densification kinetics. Using a compaction simulator, key parameters including pressure–displacement profiles, mean yield pressure, and strain rate sensitivity of the unlubricated materials were experimentally determined, while pressure transmission, residual die-wall pressure, and friction coefficient were computed. **Conclusions**: The study concluded that variations in tableting properties were primarily governed by moisture content and, for mannitol grades, by manufacturing method-dependent differences in particle microstructure. These insights provide guidance for the rational selection of polyol excipients and appropriate lubrication levels in direct compression tablet formulations.

## 1. Introduction

Polyols, such as mannitol, sorbitol, and isomalt, are widely used as excipients in direct compression tablet formulations due to their superior compaction properties [1] and their high and pH-independent solubility [2]. Various grades are available on the market, including milled and classified crystalline, granular, and spray-dried forms. The particle size, shape, and morphology of polyols determine their flowability, which is crucial for direct compression formulations and high-speed tableting processes [1,3]. As bulk sweeteners, polyols are often used at high weight fractions in compressed, orally disintegrating [4], mouth-refreshing, and chewable tablets [5]. Unlike pharmaceutical monosaccharides, such as mannitol, sorbitol, and xylitol, isomalt is a disaccharide. Its commercial grades consist of a mixture of glucose-mannitol (α-d-gluco-pyranosyl-1-6-mannitol) and glucose-sorbitol (α-d-gluco-pyranosyl-1-6-sorbitol) [6,7]. In recent years, isomalt has gained popularity in solid dosage form formulations due to its physicochemical and technological properties comparable to sorbitol and its close-to-sugar sweetness profile [8].

Due to its different solid state and morphological characteristics, the tableting process and compaction behaviour of mannitol can change drastically [1,9,10,11] and can also vary depending on the amount of lubricant [12] and the duration of lubrication [13]. Suboptimal formulations, including high levels of mannitol and lubricant, can cause tablet defects [10,14]. Additionally, the increase in the hydrophobic lubricant content reduces tablet wettability, prolongs disintegration time, and decreases the dissolution rate, which can negatively influence consumer acceptance and/or biopharmaceutical performance of tablet products.

Commonly, tablet defects are associated with high adhesion between the tablet’s radial surface and the die wall [15,16]. Upon axial compression the material densifies and undergoes irreversible deformation at compression pressure around and above the mean yield pressure (*P_y_*) [17]. This deformation usually includes particle shape changes and fragmentation. It results in changes in the specific surface area and increases the particle-to-particle bonding surface area [18], as well as the contact area between the material and die wall. The die wall is an interface between the material on one side and the compression chamber on another. The redistribution of axial compaction pressure to the radial direction can be described by the ratio of the radial to axial stress during the compression stage—the Poisson’s ratio [19,20,21,22].

The compression of the tablet is followed by decompression and relaxation, which can be characterised by the residual radial die-wall pressure [23,24]. This stage is followed by ejection. The ejection stress depends on the radial contact area, density (porosity) of the compressed material, the interaction between the material and die wall, and the residual radial die-wall pressure upon ejection [25,26].

The efficiency of the lubricant can be expected to depend on brittle fracture (opening of new unlubricated surfaces) and material behaviour upon decompression and relaxation. In turn, radial tablet pressure on the die wall should be a function of material plasticity (ability to undergo plastic deformation), applied compression pressure, punch speed, and compression pressure transmission in the radial direction.

The ejection force is recommended as an effective metric to identify and mitigate the risks of tablet defects [26], while an ejection pressure of less than 3 MPa is recommended for a successful tableting process [27]. Nowadays, the use of an instrumented die allows the measurement of the die-wall pressure, providing valuable information since high wall pressure can increase the risk of tablet radial side scratching as well as provoke tablet defects, such as capping, lamination, or chipping.

Taking into account good palatability and sweetness profiles, the high-mannitol or isomalt-loaded tablets can be considered for the preparation of low-drug-dose formulations. Thus, the investigation of the applicability of different grades of isomalt and mannitol represented by placebo tablet formulations is a meaningful task. This study aimed to investigate the effect of lubricant amount on the tableting of two grades of spray-dried mannitol (Mannogem^®^ XL Opal SD and Pearlitol^®^ 200 SD) and two grades of fluid-bed granulated isomalt (galenIQ™ 720 and galenIQ™ 721) at two levels of magnesium stearate concentrations (0.5 and 3.0 wt.%) and two levels of compaction pressures (100 and 300 MPa). Experiments were conducted using a compaction simulator and an instrumented die, considering ejection force, residual radial die-wall pressure, and other parameters.

## 2. Materials and Methods

Spray dried grades of mannitol Pearlitol^®^ 200 SD (#EQ98Q; Roquette, Lestrem, France) and Mannogem^®^ XL Opal SD (#122403728; SPI Pharma, Wilmington, NC, USA), isomalt galenIQ™ 721 (#L121390741) and galenIQ™ 720 (#L1212919U1; BENEO Palatinit GmbH, Obrigheim/Pfalz, Germany), and magnesium stearate (MgSt; #299546; Magnesia 4264; Magnesia GmbH, Lüneburg, Germany) were used in this study.

### 2.1. Loss on Drying (LoD)

Moisture content of polyols was measured by thermogravimetric analysis. The accurately weighed samples of approx. 500 mg were exposed to a constant temperature of 105 °C, and weight loss due to moisture evaporation was recorded using a Halogen Moisture Analyzer (HX204; Mettler Toledo AG, Greifensee, Switzerland). Measurements were performed in triplicate, and LoD results (%, *w*/*w*) were reported as averages ± standard deviations (S.D.) (*n* = 3) [28].

### 2.2. Karl Fischer (KF) Titration

Water content was determined by volumetric Karl Fischer titration. The accurately weighed samples of approx. 250 mg were analysed using a V10S KF titrator (Mettler Toledo AG, Bern, Switzerland) with HYDRANAL™ Methanol dry and Composite 5 reagents (Honeywell Fluka, Seelze, Germany). Triplicate measurements were performed, and the water content (%, *w*/*w*) was presented as averages ± S.D. (*n* = 3) [29].

### 2.3. Powder X-Ray Diffraction (pXRD)

pXRD analysis was conducted on a Rigaku Miniflex 600C diffractometer (Rigaku Co., Tokyo, Japan) using CuKα X-radiation (λ = 1.54182 Å) at 40 kV and 15 mA. Patterns were collected in θ/2θ geometry over the 2θ range of 3–60° with a scan rate of 5°/min at ambient temperature [30].

### 2.4. Scanning Electron Microscopy (SEM)

SEM pictures were captured with a Hitachi TM3030 microscope (Hitachi High-Tech Corp., Tokyo, Japan) at 15 kV under vacuum to assess particle morphology.

### 2.5. Specific Surface Area (SSA)

Each sample (approx. 300 mg) was weighed into a glass sample tube with a bulb volume of 1 mL and degassed under vacuum at 150 °C for 24 h using the degassing station. The samples were then transferred to the analysis port, and nitrogen gas adsorption measurements were conducted at 77 °K using a liquid nitrogen bath to maintain isothermal conditions (Nova 4200e; Quantachrome, Boynton Beach, FL, USA). The surface area was calculated by the Brunauer–Emmett–Teller (BET) method over the relative pressures of *P/P*_0_ = 0.05–0.3 using a multi-point approach with nine evenly spaced adsorption points. The t-plot method (*P/P*_0_ = 0.15–0.3) confirmed the dominant region contributing to the surface area [31].

### 2.6. Particle Size Distribution (PSD) Analysis

The PSD as well as the D_10%_, D_50%_, and D_90%_ were determined by a laser diffraction particle size analyser using an ‘Aero S’ module for dry dispersions (Mastersizer 3000, Malvern Instruments, Malvern, UK) at the specified settings: feed rate of 60%; hopper gap of 0.7–1.5 mm; air pressure of 0.8 bar. Approximately 10–15 g of the sample was used for each repetition (*n* = 3) [32].

### 2.7. Densification Profile

Densification dynamics of polyols were determined by the recording of the bulk and tapped density of the polyols. The experiment was performed using a tapped density tester (SVM II; Erweka GmbH, Langen, Germany). The measurements for samples were carried out using a 250 mL cylinder. Measurements were taken visually before any taps, as well as after 10, 110, 1110, and 3110 taps. The equipment operated at a speed of 250 taps/min and a tapping height of 3 ± 0.2 mm. The measurements were carried out three times for each sample [33].

Carr index (*Ci*) was calculated based on the bulk and tapped density (*ρ*_0_ and *ρ*_3110_; after 0 and 3110 taps, respectively) using following Equation (1):

(1) $$C_{i}=\frac{\rho_{3110}-\rho_{0}}{\rho_{3110}}\times 100\%$$

### 2.8. Mean Yield Pressure (P_y_) Determination

**Figure 1:**
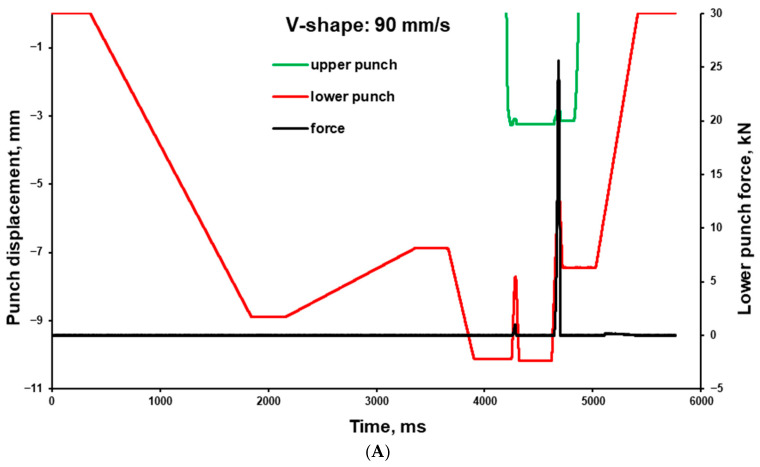

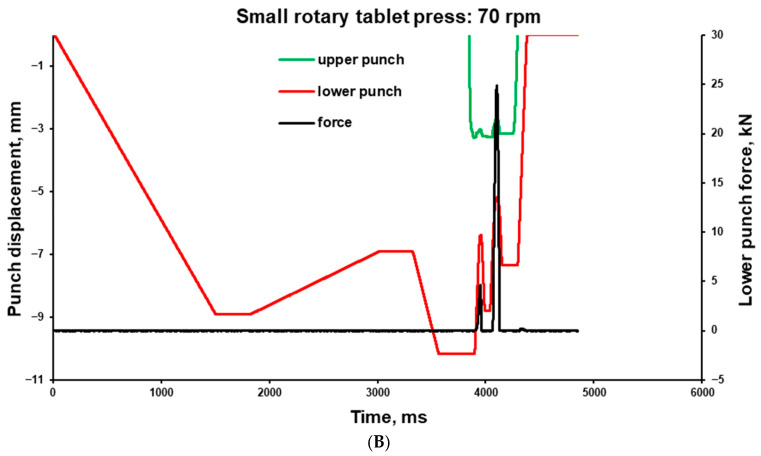
Full tableting profile illustrated with time–displacement and time–force profiles generated by compactor simulator for *P_y_* determination purpose at punch speed of 90 mm/s ((**A**) ‘V-shape’/‘saw-tooth’ compression profile) and simulation of small rotary press at speed of 70 rpm (**B**).

Unlubricated powder samples of polyols were tableted with a compaction simulator STYL’One Nano (Medelpharm, Beynost, France) using round flat Euro B punches (D = 7 mm) to obtain tablets with a target mass around 200 mg. The powder feeding into the die was performed automatically via the feed shoe. The die and punches were lubricated manually with sodium stearyl fumarate using a brush before every tableting cycle. Tableting cycles simulated by single-punch eccentric tablet press were performed at axial punch speeds of 1 and 90 mm/s (‘slow’ and ‘fast’, respectively; Figure 1A). A ‘V-shape’ (‘saw-tooth profile’) profile in accordance with the USP Pharmacopeia monograph <1062> with linear compression, and decompression phases were used. In-die P_y_ was determined automatically using the linear part of the ascending curve in the pressure range of approx. 100–350 MPa (Alix software ver. 20220711; Medelpharm, Beynost, France) as reciprocal of Heckel plot slope [34,35]. For every commercial grade of polyol, based on the preparation of ten tablets, ten single values of *P_y_* were automatically determined, and the average (Av.) and standard deviations (S.D.) were calculated.

### 2.9. Strain Rate Sensitivity (SRS)

Based on the Av. *P_y_* at punch speeds of 1 mm/s and Av. *P_y_* at punch speeds of 90 mm/s, the *SRS* (%) was calculated based on the following Equation (2) [36,37]:

(2) $$SRS\left(\%\right)=\;\frac{{Py}_{fast}-{Py}_{slow}}{{Py}_{slow}}$$

### 2.10. Sample Preparation for Tableting Process

Powder samples were prepared in the same manner. Magnesium stearate was sieved through a 0.5 mm sieve. The material was then mixed with a specific amount of magnesium stearate (0.5 or 3.0 wt.%) for 2.5 min in a double-cone blender (DVC Developer; Comasa, Barcelona, Spain) at 30 rpm. To improve microscopic homogeneity, the obtained mixture was sieved through a 1 mm sieve and mixed again for 2.5 min.

### 2.11. Tableting

Tableting cycles simulated the movement of small rotary press punches. According to STYL’One Nano presets, the simulated small rotary press had a turret diameter of 180 mm, precompression roll diameter of 44 mm, angle between rollers of 65 degrees, compression roll diameter of 160 mm, angle between main compression and the beginning of the compression ramp of 60 degrees, and an angle of ejection ramp of 20 degrees. A simulated tableting speed of 70 rpm (maximum for STYL’One Nano) was used. That corresponds to a loading, unloading, and ejection punch speed of 90 mm/s, geometric dwell time of 24 s, and relaxation time (based on the experimental data) of 170–200 ms. A pre-compression force of 5 kN (50 MPa) and compression forces of 10 and 30 kN (100 and 300 MPa) were applied (Figure 1B). Powder mixtures were tableted with round flat tooling (diameter of 11.28 mm) to obtain a target mass of 500 mg using a STYL’One Nano (Medelpharm, Beynost, France) compaction simulator. Powder feeding into the die was performed automatically via the feed shoe [38].

### 2.12. Calculated True Density

The calculated true density of the tablet composition was obtained based on the pycnometric density (*ρ*_t_) of mannitol (1.514 g/cm^3^) or isomalt (1.495 g/cm^3^ for galenIQ^™^ 720 and 1.508 g/cm^3^ for galenIQ^™^ 721), magnesium stearate (1.092 g/cm^3^) [39], and their shares (x, *w*/*w*) using the additive methodology and the following equation [40]:

$$\rho_{t}=\left(\rho_{exc1}\;\times \;x_{exc1}\;\right)+\;(\rho_{exc2}\;\times \;x_{exc2})$$

### 2.13. Apparent Density and Solid Fraction Calculation

The relative volumes and densities of the tablets were determined after ejection from the die. The apparent density (*ρ*_a_) of the tablets was calculated as the ratio of tablet weight (*w*_tab_) and volume of cylinder (based on the tablet height, *t*, and diameter, *d*) using the following equation:

$$\rho_{a}=\;\frac{w_{tab}}{\pi \;\times \;{\left(\frac{d}{2}\right)}^{2}\times \;t}$$

The solid fraction (*SF*) of the tablet was calculated using the following equation [41]:

$$SF=\;\frac{\rho_{a}}{\rho_{t}}$$

### 2.14. Residual Radial Die-Wall Pressure (P_rw_)

Considering that high ejection forces can result from high die-wall forces or friction between powder and die-wall, additional measurements were undertaken. To determine the origin of this phenomenon, radial pressure was measured at the middle of the lateral side of the tablet. Since standard compaction studies analyse the vertical forces on upper and lower punches, an instrumented die (Medelpharm, Beynost, France) was used to measure horizontal (radial) powder pressure on the die wall during compaction. This die measured residual die-wall pressure—the pressure exerted by the tablet’s radial surface on the internal die surface after removal of the upper punch and before ejection begins. Residual radial die-wall pressure was calculated as the ratio between the force applied to the die wall (*F*_die-wall_) and the radial surface area of tablet (*SA*_radial_) using the following Equation (3) [42]:

(3) $$P_{rw}=\;\frac{F_{die-wall}}{{SA}_{radial}}$$

*SA_radial_* was calculated based on the tablet thickness (distance between the punches) and the perimeter of the die.

### 2.15. Consolidation Pressure

Consolidation pressure (*P_c_*) was calculated as the average value of the maximum pressures applied on two punches using Equation (4) [23]:

(4) $$P_{c}=\;\frac{P_{upper}+\;P_{lower}}{2}$$

### 2.16. Ejection Pressure or Ejection Shear Stress (P_ej_)

The peak force during ejection by the lower punch was taken as the ejection force (*F_eject_*). Ejection pressure (*P_ej_*) was calculated based on the tablet diameter (*d_consol_*), height (*h_consol_*), and the tablet-die wall contact surface area (*π × d_consol_ × h_consol_*) under consolidation pressure (*P_consol_*) Equation (5) [43]:

(5) $$P_{ej}=\;\frac{F_{eject}}{\pi \times d_{consol}\times h_{consol}}$$

### 2.17. Pressure Transmission

Pressure transmission (*T_p_*) was calculated as the ratio of the pressure applied on the upper punch (*P_upper_*) to that on the lower punch (*P_lower_*) using Equation (6) [23]:

(6) $$T_{p}=\;\frac{P_{upper}}{P_{lower}}$$

### 2.18. Friction Coefficient (µ)

Using Coulomb’s friction law, the friction coefficient (*µ*) was calculated as the ratio of ejection pressure (*P_ej_*) to residual die-wall pressure (*P_rw_*) using Equation (7) [44]:

(7) $$\mu =\;\frac{P_{ej}}{P_{rw}}$$

### 2.19. Out-of-Die Tablet Characterisations, Radial Tablet Hardness Measurement, and Radial Tensile Strength Calculation

The out-of-die tablet thickness (*h*), diameter (*d*), and radial tablet hardness (breaking force, *F*) were measured (*n* = 10) using a tablet tester (ST50 WTDH; Sotax, Aesch, Switzerland) immediately after the compaction. Tensile strength (*τ*, MPa) was calculated using the following Equation (8) [45]:

(8) $$\tau =\;\frac{2\;F}{\pi \;d\;h}$$

### 2.20. Preparation of Dried Material

galenIQ™ 720 was dried in a drying vacuum oven (OV4-30; Jeiotech Co., Ltd., Daejeon, South Korea) at 105 °C for one day and cooled down in a desiccator with silica gel.

### 2.21. Determination of Tablet Friability

The friability test was conducted using an automatic drum tablet friability instrument (FRV 100i; Copley Scientific Ltd., Nottingham, UK) at a fixed rotation speed and test duration (25 rpm for 4 min) in accordance with European Pharmacopoeia General Monograph “Friability of Uncoated Tablets”. To measure the friability, 20 tablets (*n* = 20) were dedusted, weighed, and loaded into the testing drum. After the friability test, the tablets were dedusted, weighed, and the friability (weight loss) of tablets was calculated and expressed as a percentage of the initial weight (%) (*w*/*w*).

## 3. Results and Discussion

**Figure 2:**
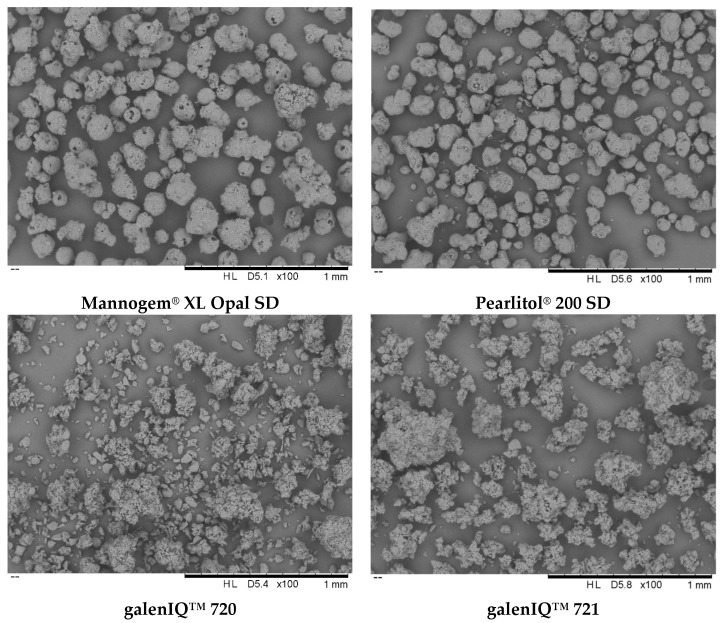
SEM of materials (magnification ×100).

Mannogem^®^ and Pearlitol^®^ particles, prepared by spray-drying of mannitol solutions, exhibit a droplet-predetermined structure, forming porous spheroids or agglomerated spheroids. In contrast, galenIQ™ 720 and galenIQ™ 721 particles, prepared by fluid-bed granulation, consist of agglomerated crystals. Isomalt (galenIQ™ 720 and galenIQ™ 721) comprises a mixture of 6-O-a-D-glucopyranosyl-D-sorbitol (1,6-GPS) and 1-O-a-D-glucopyranosyl-D-mannitol dihydrate (1,1-GPM). It is known that 1,6-GPS crystallises as anhydride, while 1,1-GPM crystallises in the hydrate form (1,1-GPM•2H_2_O). The GPM:GPS ratio is 1:1 for galenIQ™ 720 and 1:3 in galenIQ™ 721 [46]. Microscopic images of the used materials reflect their manufacturing methods (Figure 2).

**Figure 3:**
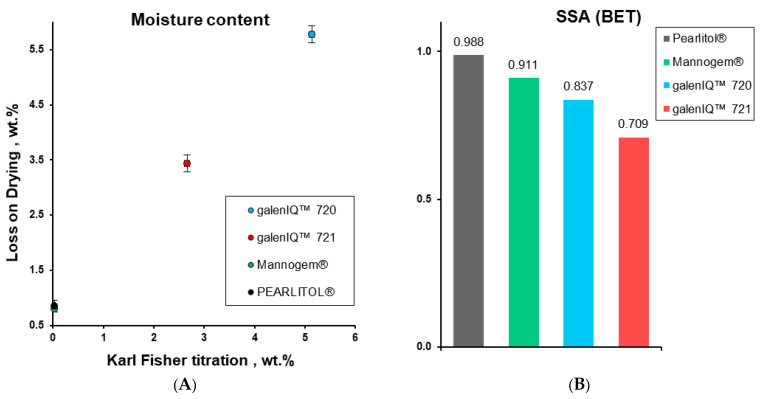
Moisture content determined by loss on drying vs. determined with Karl Fisher titration (**A**) and SSA (m^2^/g) of materials (**B**).

The moisture content can drastically influence the compaction of powders [47,48] because it can increase the rearrangement and slippage of particles [49,50] as well as decrease the hardness of crystals and increase plasticity due to intermolecular slippage [51]. Thus, moisture content in the investigated excipients was tested by two different methods, specifically loss on drying and Karl Fisher titration. Moisture content by both methods gave slightly different but comparable results (Figure 3A, Table 1). In accordance with loss on drying, the moisture content values were systematically higher than those obtained by the Karl Fisher titration method, with results of 0.80 ± 0.01, 0.85 ± 0.11, 3.44 ± 0.15, and 5.78 ± 0.08 for Mannogem^®^, Pearlitol^®^, galenIQ™ 720, and galenIQ™ 721, respectively [28].

The specific surface area of powders was measured in accordance with the BET multi-point method. Comparable but different specific surface area values decreased in a sequence of 0.988, 0.911, 0.837, and 0.709 m^2^/g for Pearlitol^®^, Mannogem^®^, galenIQ™ 720, and galenIQ™ 721, respectively (Figure 3B). Prepared by different methods, spray-dried mannitol and isomalt particles differ not only in specific surface area but also in microstructure. Microstructure differences are partially (qualitatively) revealed with microscopy (Figure 2).

**Figure 4:**
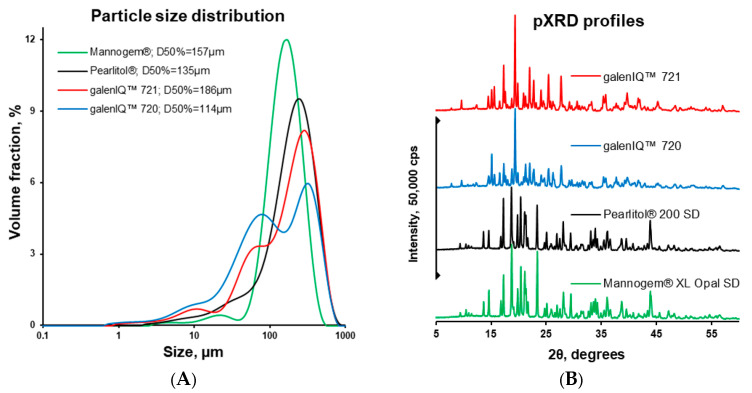
Particle size distribution (**A**) and pXRD (**B**) profiles of materials.

Particle size distribution for the investigated powders was analysed with the laser diffraction method upon dispersion in air. The D_50%_ values were found to decrease from 135 to 157 µm for Pearlitol^®^ and Mannogem^®^, and from 114 to 186 µm for galenIQ™ 720 and galenIQ™ 721 (Figure 4A).

All powders were found to have a crystal structure (Figure 4B). The pXRD profiles of the mannitol grades were considered similar. Despite the different GPM:GPS ratios in galenIQ™ 720 (1:1) and galenIQ™ 721 (1:3), their pXRD profiles were relatively similar [30,46].

**Figure 5:**
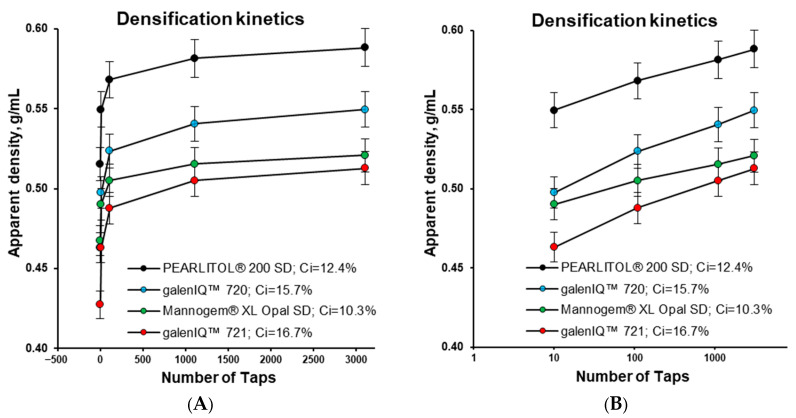
Densification profiles of materials: normal (**A**) and log-sale (**B**).

The apparent bulk and tapped densities increased in the following sequence: galenIQ™ 721, Mannogem^®^, galenIQ™ 720, and Pearlitol^®^ (Figure 5) [33]. The true densities of mannitol, galenIQ™ 721, and galenIQ™ 720 were 1.514, 1.508, and 1.495 g/cm^3^, respectively [39]. Despite its higher true density, galenIQ™ 721 demonstrated relatively lower bulk and tapped densities compared to galenIQ™ 720. Comparing the two grades of mannitol (with the same true density), Pearlitol^®^ demonstrated higher bulk and tap densities than Mannogem^®^, indicating more efficient packing. The less efficient packing of Mannogem^®^ and the higher porosity of its bulk and tapped material can be partly attributed to its more granular-like structure and/or higher particle porosity of (Figure 2). It should be noted that the first ten taps had the greatest effect on densification for all materials.

**Figure 6:**
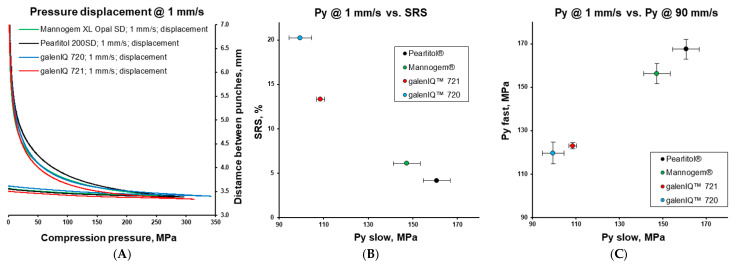
Pressure-displacement profiles (**A**), *P_y_* at slow speed vs. SRS (**B**), and *P_y_* at slow speed vs. *P_y_* at slow speed (**C**). Data was obtained for unlubricated sample but lubricated die.

V-shaped (so-called “saw-tooth”) compression cycles with linear compression and decompression at different compression speeds are widely used for powder characterisation. To determine the intrinsic properties of the materials, the investigated excipients (no admixed lubricant) were tested with a compaction simulator configured to mimic an eccentric tablet press (Figure 1) with punch speeds of 1 and 90 mm/s. Possessing close true densities, the different materials demonstrated specific pressure-displacement profiles (Figure 6A). Compared to other excipients, Pearlitol^®^ demonstrated the lowest rearrangement and the highest plastic energy. At the same time, materials demonstrated different mean yield pressures and stress rate sensitivities (Figure 6B) [52]. Morphology influences the packing of powders under pressure. In this case, Mannogem^®^ with a more developed structure demonstrated a higher rearrangement energy during compaction compared to Pearlitol^®^. Additionally, the rearrangement of particles depends on punch speed; thus, we observe higher SRS for Mannogem^®^, with a more developed structure, compared to Pearlitol^®^. The mean yield pressure at 1 mm/s increased from galenIQ^™^ 720 (99.5 MPa) to galenIQ^™^ 721 (108.6 MPa), and from Mannogem^®^ (147.3 MPa) to Pearlitol^®^ (160.8 MPa). As far as Py is a measure of material plasticity [17], it can be concluded that in this sequence, plasticity decreases from 99.5 to 160.8 MPa. It is worth noting that the mean yield pressure of galenIQ^™^ 721 (moisture content of 3.44 wt.%) was higher than that of galenIQ^™^ 720 (moisture content of 5.78 wt.%). Similarly, the mean yield pressure of mannitol (with low moisture content) was higher compared to isomalt (with relatively high moisture content) (Figure 3A and Figure 6B). In general, this result agrees with previous reports [47]. At the same time, strain rate sensitivity (1 vs. 90 mm/s) decreased in the same sequence from 20.2 to 13.3% and from 6.1 to 4.2%, respectively (Figure 6C). Among these materials, the Pearlitol^®^ mean yield pressure under axial compression was the most tablet speed independent. Chemically identical and with comparable moisture content, Pearlitol^®^ is the least plastic material of the two mannitol grades. A possible explanation could be their distinct microstructures (Figure 2), caused by different manufacturing conditions.

**Figure 7:**
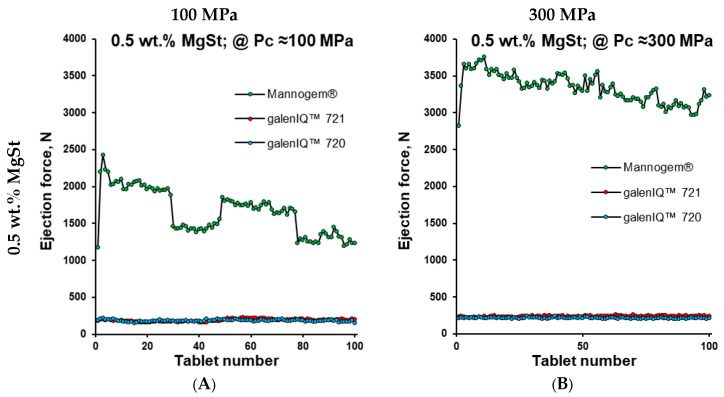

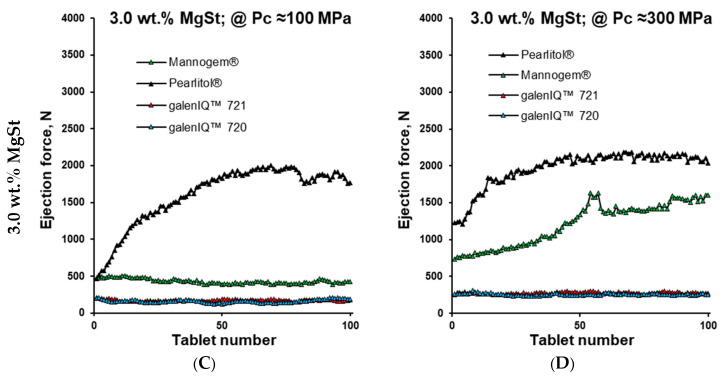
Ejection force vs. tablet number at MgSt concentration of 0.5 wt.% (**A**,**B**) and 3.0 wt.% (**C**,**D**) and consolidation pressure of 100 MPa (**A**,**C**) or 300 MPa (**B**,**D**).

The next few tests were of a practical nature. Thus, continuous tableting of 100 subsequent tablets was performed using the compactor simulator mimicking a small rotary tablet press at 70 rpm (Figure 1B). A MgSt level of 3.0 wt.% was applied to all investigated materials, and a MgSt level of 0.5 wt.% was applied to all except Pearlitol^®^. Under all conditions (MgSt levels of 0.5 and 3.0 wt.%; compaction pressures of 100 and 300 MPa), the ejection force of galenIQ™ 720 to galenIQ™ 721 tablet remained relatively low (below 300 N) and stable (Figure 7). At a MgSt level of 3.0 wt.% and compaction pressure of 100 MPa, Mannogem^®^ showed relatively low (around 500 N) and stable ejection force. Increasing compaction pressure and/or decreasing MgSt levels resulted in a drastic increase and erratic profile of the ejection force. Pearlitol^®^, tested at MgSt levels of 3.0 wt.% and at compaction pressures of 100 and 300 MPa, demonstrated gradual increase and stabilisation of ejection force around 2000 N (Figure 7C,D).

**Figure 8:**
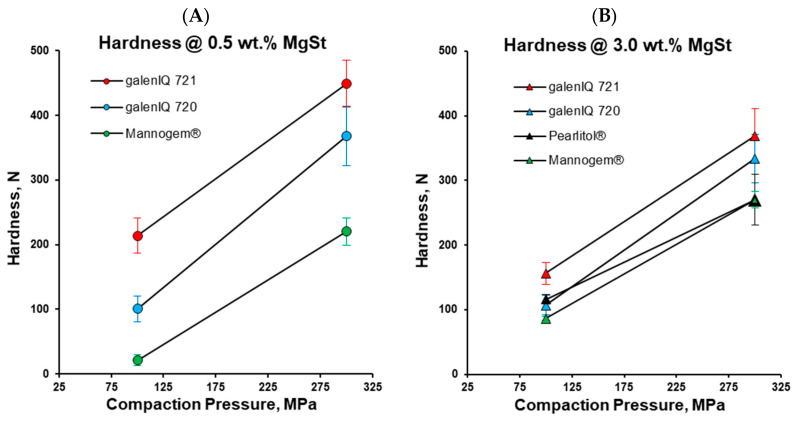
Tablet hardness as a function of consolidation pressure: at 0.5 wt.% of MgSt (**A**) and 3.0 wt.% of MgSt (**B**).

Tablet hardness at a MgSt level of 0.5 wt.% increased from Mannogem^®^ to galenIQ™ 720 to galenIQ™ 721 with an increase in compaction pressure from 100 to 300 MPa (Figure 8A). At a MgSt level of 3.0 wt.% and a compaction pressure of 100 MPa, tablet hardness values for Mannogem^®^, Pearlitol^®^, and galenIQ™ 720 were similar (around 100 N), while that of galenIQ™ 721 was significantly higher (Figure 8B). Increasing compaction pressure further increased tablet hardness, progressing from mannitol tablets to galenIQ™ 720 and then to galenIQ™ 721. Interestingly, increasing the MgSt level from 0.5 to 3.0 wt.% (Figure 8A vs. Figure 8B) at a compaction pressure of 100 MPa increased the hardness of Mannogem^®^ tablets but decreased that of galenIQ™ 721 tablets. At the same time, the apparent density and solid fraction of all tested polyols increased with increasing MgSt level and compression force (Table 2).

The friability of tablets was material-, MgSt concentration-, and compression pressure-dependent. Due to the punches used, the shape of the tablets was cylindrical, the edges were not bevelled, and thus the friability was relatively high (Table 3). In general, friability decreased (improved) with an increase in MgSt level from Pearlitol^®^ to Mannogem^®^, to galenIQ^™^ 720, and then to galenIQ^™^ 721.

To explain these practical observations, such as differences in ejection pressure, ejection pressure profile, and tablet hardness caused by MgSt level, additional experiments and computations were conducted.

**Figure 9:**
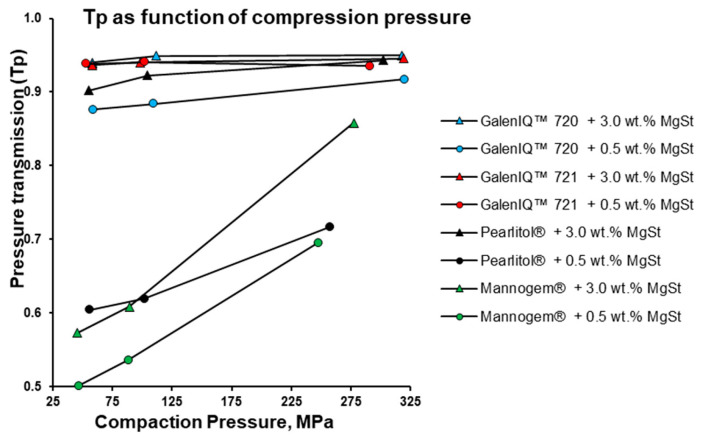
Pressure transmission (Tp) as function of compression pressure.

When mimicking a small rotary press under the used compaction simulation conditions, the upper punch remained fixed, and the compaction pressure was transmitted by the lower punch (Figure 1B). To compare the powder rheology differences, the pressure transmission ratio (*P_upper_/P_lower_*) for these materials at MgSt levels of 0.5 and 3.0 wt.% and compaction pressures of 50 (precompression), 100, and 300 MPa were tested. For galenIQ™ 721, pressure transmission was the highest (Figure 9) and almost independent of compaction pressure and MgSt level. For galenIQ™ 720, at MgSt level of 3.0 wt.%, pressure transmission was independent of consolidation pressure and nearly identical to that of galenIQ™ 721 (approx. 0.93–0.95). However, at a MgSt level of 0.5 wt.%, pressure transmission for galenIQ™ 720 was lower, and increased slightly and linearly (from 0.88 to 0.92) with increasing consolidation pressure. Pearlitol^®^, at a MgSt level of 3.0 wt.%, showed relatively high pressure transmission (close to isomalt) with a slight increase from 0.90 to 0.94 as consolidation pressure rose. At a MgSt level of 0.5 wt.%, Pearlitol^®^’s pressure transmission was lower, and increased almost linearly from 0.60 to 0.72 with consolidation pressure. The difference in pressure transmission between 3.0 and 0.5 wt.% MgSt levels was 0.30–0.23. Mannogem^®^ had the lowest pressure transmission at both MgSt levels, increasing almost linearly with consolidation pressure from 0.50 to 0.70 at 0.5 wt.% and from 0.57 to 0.86 at 3.0 wt.%. Low pressure transmission is known to correlate with inhomogeneous microscopic tablet density, solid fraction, and bonding area distribution [23]. Therefore, for Mannogem^®^, the increased pressure transmission at higher MgSt levels explains the increased tablet hardness at 100 MPa consolidation pressure (Figure 8A vs. Figure 8B).

**Figure 10:**
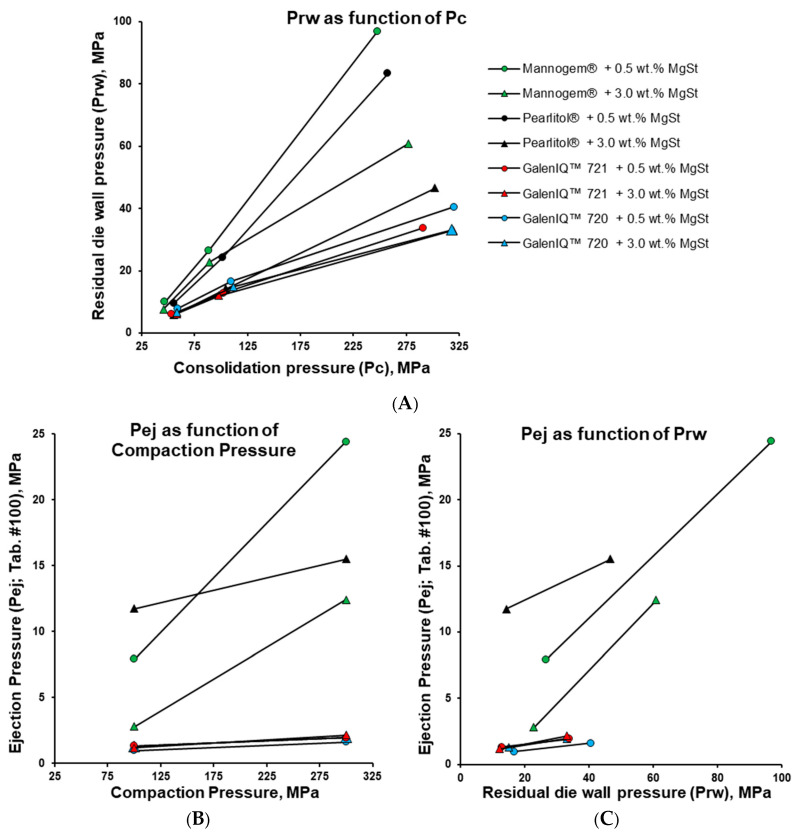
Effect of consolidation pressure on the residual die-wall pressure (**A**); ejection pressure (**B**); and effect of the residual die-wall pressure on the ejection pressure (**C**).

An instrumented die was used to explore the phenomena related to ejection force. This die measured residual die-wall pressure—the pressure exerted by the tablet’s radial surface on the internal die surface after removal of the upper punch and before ejection begins. Residual die-wall pressure increased almost linearly with consolidation pressure (Figure 10A), rising in the order of galenIQ™ 721, galenIQ™ 720, and Pearlitol^®^, and from 3.0 to 0.5 wt.% MgSt. A similar dependence on moisture content has been reported previously [47]. For all materials, residual die-wall pressure showed an almost linear relationship across consolidation pressures of 50, 100, and 300 MPa.

The ejection force of mannitol tablets varied significantly with MgSt level, consolidation pressure, and was dependent on tablet number in the sequence (Figure 7). For subsequent calculations, the ejection force of tablet #100 was used for each profile (Figure 10B,C). Considering the radial surface area, the tablet ejection force was converted into tablet ejection pressure (P_ej_), which was then plotted against the compaction pressure (Figure 10B). By analogy with the ejection force (Figure 7), the ejection pressure of galenIQ™ 721 and galenIQ™ 720 tablets was almost independent of MgSt level and compaction pressure (Figure 10B). For Mannogem^®^, the ejection pressure increased sharply with increasing consolidation pressure and with a reduction in MgSt concentration from 3.0 to 0.5 wt.%. Pearlitol^®^, at a MgSt level of 3.0 wt.%, had the highest ejection force at 100 MPa consolidation pressure but was lower than Mannogem^®^ at 300 MPa.

**Figure 11:**
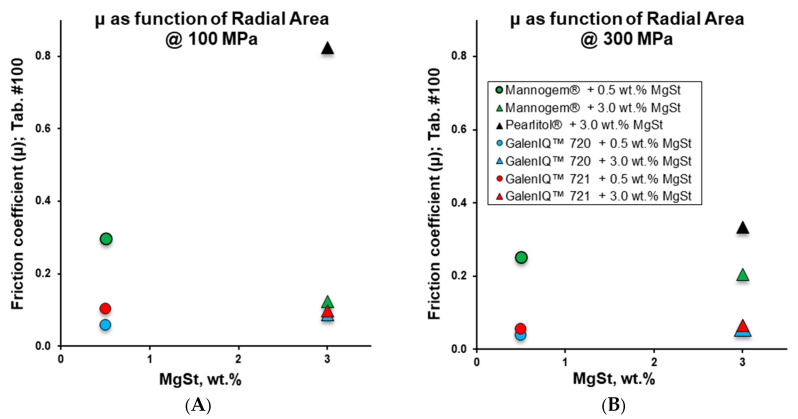
Friction coefficient as function of MgSt concentration at consolidation pressure of 100 MPa (**A**) and 300 MPa (**B**).

In the next step, ejection pressure was correlated with residual die-wall pressure (Figure 10C). In general, ejection pressure increased with residual die-wall pressure. For galenIQ™ 721 and galenIQ™ 720, ejection pressure was relatively low and almost independent of MgSt level and residual die-wall pressure. For mannitol tablets, ejection pressure increased with increasing residual die-wall pressure and decreasing MgSt concentration. The slope of Pej-Prw profiles varied between samples (Figure 10C). The friction coefficient (µ) was calculated as the ratio of Pej to Prw (Table 4), and its relationship to MgSt level was analysed (Figure 11A,B, Table 3). Increasing MgSt from 0.5 to 3.0 wt.% and compaction pressure from 100 to 300 MPa had only a slight effect on the friction coefficient of galenIQ™ 721 and 720, which remained around or below 0.1. Mannitol samples had higher friction coefficients (vs. isomalt) in all cases. Increasing the MgSt level decreased the friction coefficient of Mannogem^®^ at both compaction pressures (100 and 300 MPa), while Pearlitol^®^ had the highest friction coefficient. Interestingly, at a MgSt level of 3.0 wt.%, the friction coefficient of Mannogem^®^ was higher at 300 MPa compaction pressure. Practically, a friction coefficient above 0.2 can be lowered by increasing lubricant level; however, when the friction coefficient is near 0.1, raising the lubricant level has little effect.

Based on the mean yield pressure (*P_y_*; Figure 6) and pressure transmission (*T_p_*; Figure 9), it can be concluded that isomalt is more plastic than mannitol, possibly due to moisture content (Figure 3A). Differences between the mannitol grades likely stem from manufacturing processes, which affect particle structure and microstructure (Figure 2 and Figure 4A).

**Figure 12:**
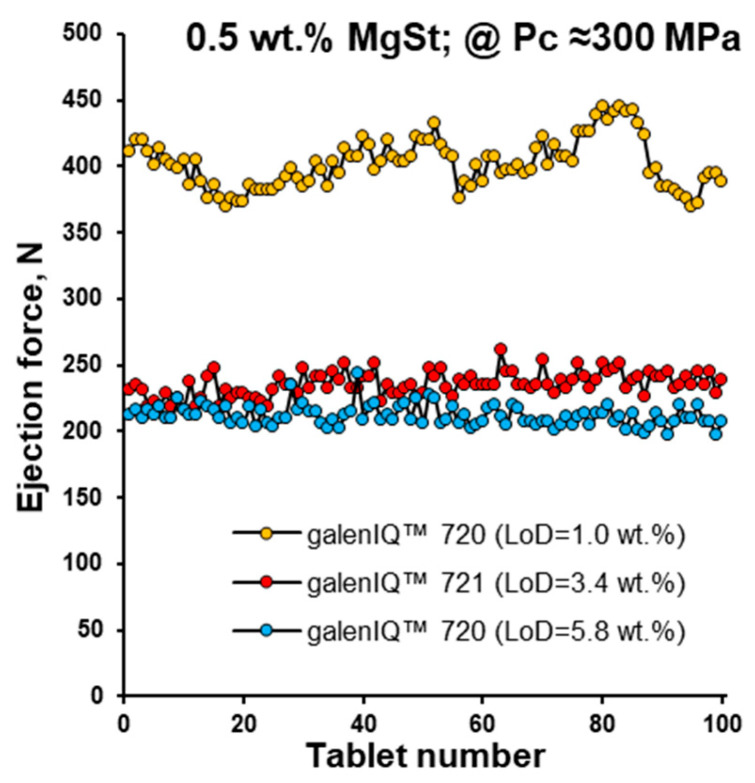
Ejection force vs. tablet number for dried galenIQ™ 720 (moisture content of 1.0 wt.%) in comparison with galenIQ™ 720 (5.8 wt.%) and galenIQ™ 721 (3.4 wt.%).

To confirm the effect of moisture content, the ejection force for dried galenIQ™ 720 (moisture content of 1.0 wt.%) was compared with galenIQ™ 720 (5.8 wt.%) and galenIQ™ 721 (3.4 wt.%). The ejection force of dried galenIQ™ 720 increased by approximately two times, and the ejection profile became more erratic (Figure 12).

## 4. Conclusions

This study contributes to the understanding of how different polyol excipients behave during direct compression tableting by investigating two levels of magnesium stearate and two compression pressures. For the first time, a compaction simulator with an instrumented die was used to compare Mannogem^®^, Pearlitol^®^, galenIQ^™^ 720, and galenIQ^™^ 721.

The difference in tableting properties, such as ejection force, pressure transmission, and residual die-wall pressure, between galenIQ^™^ 721 vs. galenIQ^™^ 720 can be attributed to their varying moisture content (3.4 vs. 5.8 wt.%) and corresponding plasticity, as reflected by mean yield pressures of 108.6 vs. 99.5 MPa, respectively. In contrast, the differences observed between Mannogem^®^ and Pearlitol^®^ cannot be explained by moisture content due to their similarity (0.80 and 0.85 wt.%, respectively). The differences are most likely due to variations in particle structure and microstructure resulting from the spray-drying process. Finally, the contrasting tableting behaviour of isomalt versus mannitol can be linked to their different moisture levels—low in mannitol (0.80–0.85 wt.%) and higher in isomalt (3.4–5.8 wt.%)—which correlates to their respective mean yield pressures. In summary, the study demonstrated distinct compaction and ejection behaviours among the investigated polyol tablet diluents, reflecting differences in deformation mechanisms, lubricant sensitivity, and overall manufacturability. Isomalt galenIQ™ 721 produced the hardest tablets and demonstrated efficient pressure transmission across all lubrication conditions. In contrast, mannitol Pearlitol^®^ required the highest lubricant level to enable smooth ejection. Notably, strong correlations between mean yield pressure and stress transmission coefficients underscores the impact of material plasticity on compaction and ejection behaviour.

The results of this study can guide the future formulations and process scale-up in terms of the desirable lubrication level for the specific polyol grades. The fluid-bed granulated moisture-containing isomalt grades demonstrated high-pressure transmission, lower ejection force, and a lower friction coefficient even at a magnesium stearate level of 0.5 wt.%. To improve the same indicators for spray-dried mannitol excipients, a relatively high level of lubrication should be used. It should be noted that pressure transmission, ejection force, friction coefficient, and related properties (such as homogeneity of microspatial density and mechanical strength) depend on tablet geometry, particularly tablet height and radial surface area. Therefore, an alternative strategy to improve these properties in mannitol tablets is to decrease tablet height and radial surface area, which can decrease the required level of lubricant.

## Figures and Tables

**Table 1:** Moisture content and particle size distribution (Av. ± S.D.).

Ingredients	Moisture Content		Particle Size Distribution		
	LoD	Karl Fisher			
	wt.%	wt.%	D_10%_, µm	D_50%_, µm	D_90%_, µm
Mannogem^®^	0.80 ± 0.00	0.028 ± 0.003	78.0 ± 0.7	157 ± 0.7	285 ± 2.4
Pearlitol^®^	0.85 ± 0.11	0.028 ± 0.002	26.3 ± 2.7	135 ± 0.6	242 ± 2.0
galenIQ^™^ 720	5.78 ± 0.15	5.133 ± 0.053	18.8 ± 1.0	114 ± 3.5	425 ± 16.9
galenIQ^™^ 721	3.44 ± 0.15	2.659 ± 0.053	31.7 ± 3.0	186 ± 4.0	423 ± 43.5

**Table 2:** Apparent density and solid fraction of prepared tablets.

Ingredients	Apparent Density, mg/mL				Solid Fraction			
	0.5 wt.% MgSt		3.0 wt.% MgSt		0.5 wt.% MgSt		3.0 wt.% MgSt	
	@100 MPa	@300 MPa	@100 MPa	@300 MPa	@100 MPa	@300 MPa	@100 MPa	@300 MPa
Mannogem^®^	1.131	1.232	1.157	1.341	0.748	0.815	0.771	0.893
Pearlitol^®^	NA	NA	1.168	1.345	NA	NA	0.778	0.896
galenIQ^™^ 720	1.252	1.439	1.254	1.416	0.839	0.954	0.845	0.955
galenIQ^™^ 721	1.211	1.418	1.219	1.417	0.804	0.942	0.815	0.948

**Table 3:** Friability of tablets (wt.%).

Ingredients	0.5 wt.% MgSt		3.0 wt.% MgSt	
	@100 MPa	@300 MPa	@100 MPa	@300 MPa
Mannogem^®^	1.55	1.68	1.32	1.01
Pearlitol^®^	NA	NA	0.90	1.32
galenIQ^™^ 720	1.09	1.34	0.92	0.81
galenIQ^™^ 721	0.60	0.69	0.82	0.30

**Table 4:** Determined values of mean yield pressure (*P_y_*) and calculated friction coefficient.

Ingredients	*P_y_* (without MgSt)		Friction Coefficient (*µ*)			
	P_y_ Slow	P_y_ Fast	0.5 wt.% MgSt		3.0 wt.% MgSt	
	MPa	MPa	@100 MPa	@300 MPa	@100 MPa	@300 MPa
Mannogem^®^	147.3	156.3	0.298	0.252	0.123	0.204
Pearlitol^®^	160.8	167.5	NA	NA	0.825	0.333
galenIQ^™^ 720	99.5	119.6	0.060	0.040	0.087	0.059
galenIQ^™^ 721	108.6	123.0	0.104	0.057	0.097	0.065

## Data Availability

The raw data supporting the conclusions of this article will be made available by the correspondence author on request.
